# Supplementary material for: MetaRibo-Seq measures translation in microbiomes
Source: Nat Commun. 2020 Jun 29;11:3268. doi: 10.1038/s41467-020-17081-z (PMC7324362; doi:10.1038/s41467-020-17081-z)
Supplement: Supplementary file 10 — Supplementary Data 7 [file 41467_2020_17081_MOESM10_ESM.zip › File2/Confidence_High_Taxonomy/394879_out.krona.html]

Javascript must be enabled to view this page.

members
magnitude
magnitudeUnassigned
count
unassigned
taxon
rank

394879\_out

439

superkingdom
2
439

976
439
phylum

class
439
200643

order
439
171549

815
435
family

816
6
435

SRS015960\_contig\_number\_10753SRS021948\_contig\_number\_11363SRS048870\_contig\_number\_11026SRS1055043\_contig\_number\_18861SRS1055067\_contig\_number\_9230SRS893270\_contig\_number\_12459
genus

1
162156
species

SRS024492\_contig\_number\_15775


SRS016132\_contig\_number\_1073SRS075341\_contig\_number\_15066
species
2
246787

671267
8

SRS013687\_contig\_number\_31723SRS016381\_contig\_number\_2871SRS024388\_contig\_number\_1719SRS053356\_contig\_number\_43404SRS065504\_contig\_number\_22144SRS1041132\_contig\_number\_7528SRS150029\_contig\_number\_2456SRS893358\_contig\_number\_10698
species


SRS015794\_contig\_number\_1299SRS017821\_contig\_number\_23409SRS018575\_contig\_number\_5831SRS018984\_contig\_number\_3645SRS024625\_contig\_number\_8732SRS077024\_contig\_number\_11567SRS078419\_contig\_number\_18137SRS104693\_contig\_number\_2290SRS1055022\_contig\_number\_16807SRS143372\_contig\_number\_6718
species
818
10

1
28111

SRS021219\_contig\_number\_2261
species

1638780
1
species

SRS013476\_contig\_number\_36255

species

SRS011084\_contig\_number\_15961SRS011134\_contig\_number\_16141SRS012969\_contig\_number\_21313SRS013521\_contig\_number\_11182SRS014235\_contig\_number\_24993SRS014459\_contig\_number\_9710SRS014683\_contig\_number\_7311SRS015264\_contig\_number\_10068SRS015431\_contig\_number\_38246SRS016095\_contig\_number\_25528SRS017701\_contig\_number\_11556SRS018817\_contig\_number\_3459SRS018888\_contig\_number\_6789SRS019068\_contig\_number\_48160SRS019161\_contig\_number\_25996SRS022609\_contig\_number\_16995SRS023526\_contig\_number\_17262SRS023583\_contig\_number\_4898SRS043701\_contig\_number\_14621SRS043701\_contig\_number\_16829SRS044535\_contig\_number\_18134SRS049896\_contig\_number\_11849SRS049959\_contig\_number\_contig-100\_813.247587SRS049995\_contig\_number\_12525SRS050752\_contig\_number\_19909SRS050925\_contig\_number\_16166SRS056259\_contig\_number\_26244SRS056259\_contig\_number\_28828SRS063985\_contig\_number\_4842SRS064557\_contig\_number\_2981SRS074670\_contig\_number\_6346SRS075398\_contig\_number\_contig-100\_900.21661SRS075963\_contig\_number\_contig-100\_645.645SRS077294\_contig\_number\_12114SRS077454\_contig\_number\_5621SRS077849\_contig\_number\_7181SRS078242\_contig\_number\_11767SRS101433\_contig\_number\_2868SRS104036\_contig\_number\_contig-100\_1694.55064SRS1041031\_contig\_number\_27393SRS1041095\_contig\_number\_3513SRS1041129\_contig\_number\_contig-100\_2092.82142SRS1041140\_contig\_number\_22890SRS1041157\_contig\_number\_6293SRS104975\_contig\_number\_2201SRS105153\_contig\_number\_14439SRS1055069\_contig\_number\_9841SRS143181\_contig\_number\_5893SRS147346\_contig\_number\_28121SRS149181\_contig\_number\_15002SRS893252\_contig\_number\_12840SRS893369\_contig\_number\_2SRS893382\_contig\_number\_3645
1946084
53

204516
2
species

SRS013800\_contig\_number\_11260SRS052027\_contig\_number\_11456

species

SRS050925\_contig\_number\_13971
2292943
1


SRS018836\_contig\_number\_15337SRS020328\_contig\_number\_13927
species
2292944
2

species

SRS103987\_contig\_number\_5512
1
2292941

1
29523

SRS047433\_contig\_number\_947
species

species

SRS044535\_contig\_number\_364
1
2292939


SRS012273\_contig\_number\_23050SRS013098\_contig\_number\_12432SRS013940\_contig\_number\_20209SRS016018\_contig\_number\_7459SRS017821\_contig\_number\_10536SRS018049\_contig\_number\_1067SRS018133\_contig\_number\_9506SRS018541\_contig\_number\_9899SRS019286\_contig\_number\_5695SRS019685\_contig\_number\_16312SRS023346\_contig\_number\_684SRS024132\_contig\_number\_21047SRS024435\_contig\_number\_33625SRS024549\_contig\_number\_1224SRS024663\_contig\_number\_4645SRS042628\_contig\_number\_30099SRS045195\_contig\_number\_2296SRS045713\_contig\_number\_13321SRS046717\_contig\_number\_596SRS048981\_contig\_number\_2848SRS054590\_contig\_number\_1039SRS058070\_contig\_number\_5501SRS058770\_contig\_number\_19855SRS062701\_contig\_number\_4006SRS064757\_contig\_number\_17042SRS075021\_contig\_number\_2081SRS077231\_contig\_number\_8283SRS098655\_contig\_number\_13560SRS098717\_contig\_number\_6909SRS101268\_contig\_number\_1568SRS1041112\_contig\_number\_482SRS1041145\_contig\_number\_31299SRS104636\_contig\_number\_14072SRS1055034\_contig\_number\_419SRS140645\_contig\_number\_1288SRS142712\_contig\_number\_5383SRS148874\_contig\_number\_1909SRS149784\_contig\_number\_24858SRS149879\_contig\_number\_25864SRS893230\_contig\_number\_2057SRS971276\_contig\_number\_9160
species
41
357276

species

SRS013215\_contig\_number\_413SRS020622\_contig\_number\_2807SRS046369\_contig\_number\_15169SRS056519\_contig\_number\_1933SRS097920\_contig\_number\_11899SRS893256\_contig\_number\_1333
28116
6


SRS053214\_contig\_number\_6529
species
371601
1


SRS147139\_contig\_number\_27318
species
1
1078089


SRS012902\_contig\_number\_952SRS015578\_contig\_number\_8819SRS050941\_contig\_number\_11868SRS051610\_contig\_number\_1535SRS057717\_contig\_number\_5801SRS064276\_contig\_number\_16272SRS147557\_contig\_number\_29050SRS893342\_contig\_number\_2430
species
8
817

47678
3

SRS048164\_contig\_number\_6114SRS052078\_contig\_number\_989SRS098827\_contig\_number\_4092
species

species

SRS065176\_contig\_number\_7183
387090
1

266
821

SRS011239\_contig\_number\_2166SRS011302\_contig\_number\_12401SRS011405\_contig\_number\_3075SRS013382\_contig\_number\_1165SRS013638\_contig\_number\_9210SRS013951\_contig\_number\_28273SRS013965\_contig\_number\_27527SRS014287\_contig\_number\_2894SRS014313\_contig\_number\_851SRS014412\_contig\_number\_17252SRS014534\_contig\_number\_contig-100\_276.14361SRS014736\_contig\_number\_contig-100\_1008.63705SRS014855\_contig\_number\_3700SRS014923\_contig\_number\_29154SRS015065\_contig\_number\_35659SRS015190\_contig\_number\_10355SRS015217\_contig\_number\_12879SRS015486\_contig\_number\_1050SRS015579\_contig\_number\_14560SRS015663\_contig\_number\_20464SRS015694\_contig\_number\_4811SRS015782\_contig\_number\_26165SRS015854\_contig\_number\_3139SRS016056\_contig\_number\_3733SRS016203\_contig\_number\_contig-100\_227.74649SRS016335\_contig\_number\_43238SRS016753\_contig\_number\_1245SRS016840\_contig\_number\_2791SRS016897\_contig\_number\_contig-100\_773.22342SRS016954\_contig\_number\_11860SRS017103\_contig\_number\_22431SRS017191\_contig\_number\_12041SRS017247\_contig\_number\_9617SRS017307\_contig\_number\_18526SRS017375\_contig\_number\_102SRS017433\_contig\_number\_9296SRS017521\_contig\_number\_18472SRS017745\_contig\_number\_662SRS017859\_contig\_number\_3817SRS018351\_contig\_number\_18349SRS018427\_contig\_number\_22222SRS018623\_contig\_number\_12407SRS018936\_contig\_number\_9473SRS019030\_contig\_number\_2858SRS019178\_contig\_number\_1505SRS019267\_contig\_number\_1309SRS019381\_contig\_number\_13977SRS019397\_contig\_number\_1136SRS019445\_contig\_number\_31275SRS019496\_contig\_number\_11410SRS019601\_contig\_number\_3518SRS019601\_contig\_number\_12348SRS019693\_contig\_number\_6982SRS019808\_contig\_number\_contig-100\_813.112748SRS019968\_contig\_number\_12608SRS020233\_contig\_number\_44737SRS020394\_contig\_number\_10276SRS020508\_contig\_number\_227SRS021153\_contig\_number\_1025SRS021484\_contig\_number\_6400SRS022071\_contig\_number\_23992SRS022137\_contig\_number\_10530SRS022524\_contig\_number\_14613SRS022524\_contig\_number\_15473SRS023829\_contig\_number\_686SRS023914\_contig\_number\_15026SRS023971\_contig\_number\_675SRS024009\_contig\_number\_2846SRS024075\_contig\_number\_9343SRS024331\_contig\_number\_12644SRS043001\_contig\_number\_5825SRS043411\_contig\_number\_11956SRS043667\_contig\_number\_2190SRS043768\_contig\_number\_23833SRS045004\_contig\_number\_956SRS045244\_contig\_number\_2492SRS045645\_contig\_number\_13902SRS045739\_contig\_number\_1859SRS046068\_contig\_number\_2220SRS046502\_contig\_number\_13664SRS046810\_contig\_number\_contig-100\_39.40SRS047014\_contig\_number\_38316SRS047044\_contig\_number\_contig-100\_701.81424SRS049402\_contig\_number\_8980SRS049446\_contig\_number\_134SRS049712\_contig\_number\_24291SRS049773\_contig\_number\_28531SRS049900\_contig\_number\_15035SRS050026\_contig\_number\_8311SRS050299\_contig\_number\_9872SRS050422\_contig\_number\_10086SRS050520\_contig\_number\_contig-100\_428.21813SRS050801\_contig\_number\_contig-100\_236.35698SRS050974\_contig\_number\_798SRS050998\_contig\_number\_13173SRS051031\_contig\_number\_13648SRS051882\_contig\_number\_11321SRS052576\_contig\_number\_contig-100\_336.337SRS053087\_contig\_number\_2772SRS053335\_contig\_number\_contig-100\_560.109134SRS053398\_contig\_number\_1023SRS053649\_contig\_number\_4315SRS054059\_contig\_number\_1642SRS054352\_contig\_number\_3129SRS054905\_contig\_number\_20547SRS055017\_contig\_number\_25163SRS055966\_contig\_number\_1089SRS056273\_contig\_number\_24043SRS056537\_contig\_number\_47873SRS057478\_contig\_number\_5066SRS057480\_contig\_number\_contig-100\_170.68272SRS058268\_contig\_number\_46SRS058723\_contig\_number\_1834SRS062427\_contig\_number\_776SRS062654\_contig\_number\_17658SRS063127\_contig\_number\_1603SRS063190\_contig\_number\_14280SRS063370\_contig\_number\_1200SRS063489\_contig\_number\_2254SRS063813\_contig\_number\_4912SRS063928\_contig\_number\_45SRS064232\_contig\_number\_2409SRS064645\_contig\_number\_1238SRS065176\_contig\_number\_10992SRS065397\_contig\_number\_3930SRS074586\_contig\_number\_5441SRS074964\_contig\_number\_89SRS075078\_contig\_number\_13523SRS075716\_contig\_number\_4425SRS075821\_contig\_number\_23324SRS075878\_contig\_number\_10699SRS075984\_contig\_number\_17595SRS076756\_contig\_number\_14128SRS076876\_contig\_number\_contig-100\_891.17521SRS076976\_contig\_number\_2871SRS077127\_contig\_number\_10506SRS077194\_contig\_number\_12426SRS077231\_contig\_number\_9825SRS077335\_contig\_number\_5067SRS077392\_contig\_number\_31475SRS077502\_contig\_number\_14912SRS077552\_contig\_number\_14153SRS077641\_contig\_number\_19889SRS077730\_contig\_number\_16586SRS077991\_contig\_number\_contig-100\_226.227SRS078176\_contig\_number\_10543SRS078665\_contig\_number\_3714SRS097889\_contig\_number\_4400SRS097958\_contig\_number\_20313SRS098073\_contig\_number\_4437SRS098571\_contig\_number\_17468SRS098644\_contig\_number\_3178SRS098827\_contig\_number\_4115SRS100021\_contig\_number\_3559SRS100907\_contig\_number\_3225SRS101376\_contig\_number\_7153SRS104084\_contig\_number\_13047SRS1041032\_contig\_number\_11237SRS1041036\_contig\_number\_1483SRS1041037\_contig\_number\_2477SRS1041037\_contig\_number\_15839SRS1041038\_contig\_number\_15634SRS1041039\_contig\_number\_9743SRS1041090\_contig\_number\_5322SRS1041091\_contig\_number\_5624SRS1041092\_contig\_number\_5746SRS1041116\_contig\_number\_9157SRS1041118\_contig\_number\_11275SRS1041130\_contig\_number\_2682SRS1041133\_contig\_number\_28566SRS1041134\_contig\_number\_10454SRS1041136\_contig\_number\_11649SRS1041137\_contig\_number\_17134SRS1041138\_contig\_number\_2554SRS1041139\_contig\_number\_2852SRS1041141\_contig\_number\_contig-100\_722.83761SRS1041142\_contig\_number\_3574SRS1041143\_contig\_number\_6572SRS1041144\_contig\_number\_3324SRS1041147\_contig\_number\_1303SRS104327\_contig\_number\_2923SRS104400\_contig\_number\_20493SRS104485\_contig\_number\_1007SRS104693\_contig\_number\_8629SRS104912\_contig\_number\_contig-100\_97.112686SRS105082\_contig\_number\_18933SRS1054716\_contig\_number\_10393SRS1054928\_contig\_number\_1046SRS1055038\_contig\_number\_1438SRS1055049\_contig\_number\_10119SRS1055056\_contig\_number\_contig-100\_213.66813SRS1055076\_contig\_number\_2497SRS1055083\_contig\_number\_374SRS140492\_contig\_number\_30984SRS140513\_contig\_number\_1487SRS142503\_contig\_number\_28420SRS142542\_contig\_number\_22802SRS142781\_contig\_number\_3592SRS142781\_contig\_number\_7381SRS142890\_contig\_number\_32085SRS142923\_contig\_number\_18549SRS142980\_contig\_number\_1240SRS143085\_contig\_number\_2532SRS143148\_contig\_number\_13884SRS143342\_contig\_number\_14754SRS143417\_contig\_number\_27066SRS143523\_contig\_number\_14052SRS143598\_contig\_number\_11550SRS143722\_contig\_number\_8738SRS143780\_contig\_number\_8976SRS143895\_contig\_number\_39437SRS143991\_contig\_number\_19659SRS144135\_contig\_number\_24653SRS144183\_contig\_number\_31951SRS144297\_contig\_number\_9313SRS144362\_contig\_number\_24156SRS144506\_contig\_number\_27897SRS144537\_contig\_number\_contig-100\_2257.268753SRS144603\_contig\_number\_11594SRS144714\_contig\_number\_509SRS144753\_contig\_number\_11897SRS145308\_contig\_number\_758SRS146764\_contig\_number\_16775SRS146813\_contig\_number\_21216SRS147022\_contig\_number\_1179SRS147039\_contig\_number\_2753SRS147377\_contig\_number\_10282SRS147425\_contig\_number\_8388SRS147614\_contig\_number\_10036SRS147653\_contig\_number\_6620SRS147766\_contig\_number\_14773SRS147977\_contig\_number\_10573SRS148091\_contig\_number\_2678SRS148159\_contig\_number\_4963SRS148196\_contig\_number\_38297SRS148253\_contig\_number\_4430SRS148319\_contig\_number\_4633SRS148424\_contig\_number\_25334SRS148511\_contig\_number\_12462SRS148721\_contig\_number\_30761SRS148784\_contig\_number\_14557SRS148817\_contig\_number\_9447SRS148970\_contig\_number\_1157SRS149075\_contig\_number\_11015SRS149244\_contig\_number\_4216SRS149325\_contig\_number\_10249SRS892882\_contig\_number\_11973SRS893170\_contig\_number\_1424SRS893172\_contig\_number\_9032SRS893187\_contig\_number\_10494SRS893253\_contig\_number\_976SRS893259\_contig\_number\_4070SRS893279\_contig\_number\_16701SRS893285\_contig\_number\_981SRS893288\_contig\_number\_802SRS893292\_contig\_number\_2856SRS893295\_contig\_number\_4348SRS893300\_contig\_number\_2390SRS893327\_contig\_number\_2171SRS893341\_contig\_number\_2224SRS893366\_contig\_number\_8713SRS893373\_contig\_number\_21363SRS893378\_contig\_number\_7190SRS893380\_contig\_number\_246SRS893383\_contig\_number\_18328SRS971275\_contig\_number\_34828
species

1
1263056

SRS1055095\_contig\_number\_2022
species

species

SRS064973\_contig\_number\_19
2292955
1

820
3

SRS016495\_contig\_number\_19215SRS042284\_contig\_number\_4352SRS1041144\_contig\_number\_1710
species

species

SRS015794\_contig\_number\_991SRS022713\_contig\_number\_1638SRS023715\_contig\_number\_17709SRS042966\_contig\_number\_11608SRS075773\_contig\_number\_30845SRS098881\_contig\_number\_14459SRS143070\_contig\_number\_2126SRS143876\_contig\_number\_3448
8
46506

5
2292935
species

SRS014613\_contig\_number\_12353SRS048060\_contig\_number\_7579SRS064276\_contig\_number\_34741SRS1054691\_contig\_number\_15221SRS1055099\_contig\_number\_11706

family
171552
1

genus
838
1

1
1262925
species

SRS049995\_contig\_number\_29606

family
3
171550


SRS056259\_contig\_number\_20823
genus
1
239759
3

species

SRS045826\_contig\_number\_19390
1896968
1


SRS098061\_contig\_number\_11805
species
1
28117
